# Determinants of an evidence-based practice environment: an interpretive description

**DOI:** 10.1186/s43058-020-00070-0

**Published:** 2020-10-06

**Authors:** Jed Duff, Laura Cullen, Kirsten Hanrahan, Victoria Steelman

**Affiliations:** 1 Queensland University of Technology, Nursing & Midwifery Research Centre, Royal Brisbane & Women’s Hospital, Butterfield St, Herston, QLD 4029 Australia; 2 University of Newcastle, School of Nursing and Midwifery, University Drive, Callaghan, NSW 2308 Australia; 3 University of Iowa Hospitals and Clinics, Nursing Research and Evidence-Based Practice, 200 Hawkins Dr, Iowa City, IA 52242 USA; 4 College of Nursing, University of Iowa, 50 Newton Rd, Iowa City, IA 52242 USA

## Abstract

**Background:**

Despite the available research to inform nursing practice, many patients still fail to receive evidence-based care. Several evidence-based practice (EBP) models have been developed to guide nurses through the steps in the process, yet these models have not been uniformly adopted or consistently used. The original purpose of this research was to gather perspectives and experiences of nurses using the Iowa Model of EBP to help inform its introduction into other practice settings. As a more in-depth understanding was gained, the emphasis of the study shifted towards understanding the determinants of the EBP environment.

**Method:**

The study was conducted in an 800-bed comprehensive academic medical centre in the USA with a 25-year history of using the Iowa Model of EBP. Semi-structured in-depth interviews were conducted with twelve nurses from various roles to ascertain their perspectives and experiences using the model. The interview transcripts were reviewed alongside relevant published literature and internal documents in a process of synthesising, theorising, and conceptualising. Data were collected during the first half of 2019.

**Results:**

Four determinants of the local EBP environment were identified from the perspectives and experiences of participants: (1) the importance of a shared model to guide staff through the EBP process; (2) support for EBP in the form of education, hands-on training, and knowledge infrastructure; (3) active team facilitation by direct care nurses, nurse managers, nurse specialists, and nurse scientists; and (4) a culture and leadership that encourages EBP.

**Conclusion:**

Introducing an EBP model is an essential first step for an organisation to improve consistent and reliable evidence-based care; to be most effective, this should be done in conjunction with efforts to optimise the EBP environment.

Contributions to the literature

- The findings from this research support the idea that EBP is most effective in a supportive practice environment.
- A supportive practice environment includes EBP education and training, team facilitation, and a supportive culture and leadership.
- Organisations wishing to implement an EBP model should do so in conjunction with efforts to optimise the practice environment.

## Background

Evidence-based practice (EBP) is considered the gold standard of care, and as such, it is now an expectation of patients, regulatory agencies, and healthcare funders. Despite the abundance of research to inform clinical practice, many patients still fail to receive evidence-based care. Population-level estimates of the quality of health care are limited, but two landmark studies, one from the USA [1] and one from Australia [2], estimate adherence to clinical practice guidelines at 55% and 57%, respectively. Both studies audited a nationwide random sample of medical records to compare the care delivered with nationally endorsed guidelines. The studies found that almost four out of every ten people do not get evidence-based care, or worse still, get care that is known to be ineffective, or even harmful [1, 2].

There have been many reasons put forward as to why it is so challenging to provide evidence-based care. One of the most obvious is the fact that new evidence is being generated at an ever-increasing rate. It is estimated that nearly one million new articles are posted on PubMed annually [3]. Healthcare professionals face the challenge of providing care while also finding, appraising, and integrating new evidence into their routine practice. Unfortunately, for many clinicians, the environments they work in are not always conducive to this [4].

Several models have been developed to guide nurses through the steps necessary for EBP [5]. Although they vary in explicit criteria, they generally all contain a familiar series of steps from the identification of a clinical problem, to evidence synthesis, and then implementation and evaluation [6]. In contrast to evidence-based medicine, which is primarily focused on the clinician-patient level, EBP models focus on integrating evidence at a systems level [7]. The Iowa Model of EBP (Iowa Model) [8] is one of the most widely used in the USA. The model was developed 25 years ago by nurses at the University of Iowa Hospital and faculty from the University of Iowa College of Nursing [9]. The model underwent a significant review and revision in 2017 [8].

While many of the EBP models have existed for two decades or more, their use varies considerably among organisations and between countries which has led to calls for more widespread dissemination and adoption [10]. The original aim of this research was to gather the perspectives and experiences of nurses using the Iowa Model to inform its introduction to other practice settings. As with many interpretive descriptive studies, the focus of the research departed slightly from its original aim [11]. As a more in-depth understanding was gained, the focus broadened to include all the determinants of the EBP environment.

## Method

### Design

An interpretive descriptive methodology [12] was used to identify themes and patterns within the subjective perspectives and experiences of nurses using the Iowa Model.

### Analytic framework

In interpretive description, qualitative inquiry is located within the existing body of knowledge with themes and subthemes constructed through thoughtful linkages with other work in the field [13, 14]. The current research on EBP and the models, frameworks, and theories that endeavour to explain it have informed this study. Specifically, process theories that provide practical guidance for planning and executing implementation, and determinant frameworks, which specify constructs that influence or predict implementation outcomes [15]. The study was also informed by the two decades of research on the nursing practice environment [16], which is defined as the organisational characteristics in the work environment that make professional practice easier or more difficult.

### Setting

The setting was an 800-bed comprehensive academic medical centre and level one trauma centre located in the Midwestern USA. The hospital has Magnet designation—recognition of nursing excellence, quality patient care, and innovation in professional nursing practice [17]. There are over 13,000 employees, including 3000 professional nurses who care for 37,000 in-patients, over a million clinic visits and 58,000 emergency department visits annually. The Iowa Model was first developed by local clinicians and nursing faculty 25 years ago, and it has been used and improved on ever since.

### Participants

An email invitation was sent by an administrative assistant from the Office of Nursing Research and EBP to nurses known to have experience using the Iowa Model. Maximum variation sampling [18] was employed to target nurses with different roles. The intention was to gather a broad selection of experiences and perspectives. All but one person accepted the invitation to participate. In keeping with the methodology [12], data saturation was not the desired outcome as it was acknowledged that there might be an infinite variety of perspectives and experiences. Instead, the focus was to interview participants until a deep understanding was obtained while recognising that outliers may still exist.

### Data collection and analysis

A semi-structured interview schedule was developed by the researchers (supplementary material). A reflective researcher diary documenting observations and experiences was used contemporaneously [19]. Interviews were conducted by the lead author, who was embedded in the organisation as part of an academic exchange program. This extended exposure helped him develop insights into the practice setting and build trust and rapport with the participants [20]. The interviews were conducted in the office of the lead author or the participant’s office. Interviews were audio-recorded and transcribed verbatim. Documents relevant to the organisation’s EBP program were reviewed to generate further insights and corroborate the interview data [21]. The documents reviewed included annual reports, accreditation materials, meeting minutes, peer-reviewed publications, organisational webpages, and EBP training materials. The published works that inform theme development have been referenced in the appropriate section of the findings.

Data analysis was an ongoing iterative process conducted throughout data collection by the first and last author [12]. All transcribed interviews were uploaded to NVivo 12 software where they were read in detail several times. This process enabled the identification of similarities and differences between participants, making it possible to see patterns and generate initial themes. The review of the transcripts was interspersed with strategic periods of immersion in the literature and internal documents as part of the process of synthesising, theorising, and conceptualising [14]. After a preliminary analysis was performed, the initial themes and organising framework were discussed with participants and other stakeholders [11] including nurse leaders, academics, and direct care staff. All feedback was incorporated into further rounds of synthesising, theorising, and conceptualising [14].

The practices recommended by Thorne et al. [14] were implemented to maintain rigour: specifically, prolonged engagement with participants, the use of a reflexive researcher diary, the triangulation of data from multiple sources, and the confirmation of initial themes and organising framework with participants. The study has been reported using the consolidated criteria for reporting qualitative research [22].

## Results

A total of twelve in-depth interviews lasting between 60 and 120 min were conducted between February and May 2019. Participants included three staff nurses, three nurse faculty, one nurse leader, two nurse scientists, two nurse specialists, and one nurse manager. The three staff nurses had 2 to 8 years’ experience using the Iowa Model while the other participants had more than 20 years (see Table 1).

**Table 1:** Characteristics of participants

Participant	Role	Experience with the Iowa Model
1	Staff nurse	2 years
2	Staff nurse	2 years
3	Staff nurse	8 years
4	Nurse faculty	25 years
5	Nurse faculty	25 years
6	Nurse faculty	25 years
7	Nurse scientist	25 years
8	Nurse scientist	25 years
9	Nurse leader	25 years
10	Nurse specialist	20 years
11	Nurse specialist	25 years
12	Nurse manager	25 years

**Fig. 1:**
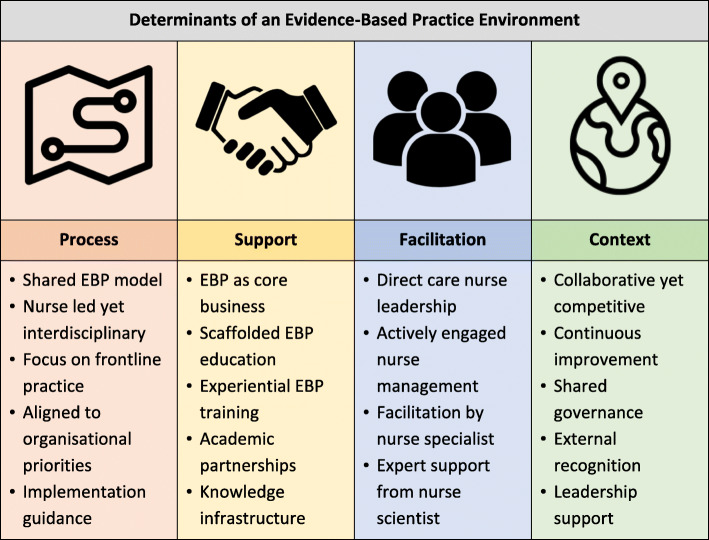
Determinants of the EBP environment

As the interviews and analysis progressed, it became apparent that a supportive practice environment was the primary influence on nurses’ ability to consistently and effectively deliver evidence-based care. Determinants of the EBP environment were clustered into four themes: process, support, facilitation, and context. These themes are represented in Fig. 1 and described in detail below.

### Process

All participants agreed that having a shared EBP model to guide the process was a key determinant of the EBP environment. More than one interviewee described the model as a ‘roadmap’ that staff use to navigate the EBP process.

It [the Iowa Model] is our roadmap for EBP. The model helps us know if you’re heading in the right direction, where you’re at, where you’re going. When you get off course, it helps to pull you back on course. It helps identify some of the hazards and potholes that we can expect and how to troubleshoot through them. P7

The fact that the model was locally developed was a benefit as it gave staff a sense of ownership and an investment in the EBP process. Participants did acknowledge that the Iowa Model was one of several EBP process models that contained very similar steps. *‘Having a model, whichever one is used’,* was the essential first step in creating an EBP environment.

The Iowa Model was first developed 25 years ago by local nurses, and it is still nursing led, yet interdisciplinary. It was pointed out that one step of the model is the establishment of an appropriate team to address the practice issue, and in almost all cases, this involved interdisciplinary collaboration. Interviewees saw the fact that EBP is chiefly led by nursing as entirely appropriate.

*I’m comfortable with EBP primarily being led by nursing because nursing is at the front line of care. It’s good to have one discipline who’s leading it [EBP], who really gets the process, can establish the infrastructure, and hardwired it into business as usual. They can then partner with other professions to collaborate. P10*

An identified strength of the Iowa Model and a proposed reason for its success is its focus on frontline practice issues that are meaningful to staff and patients. Projects, where the topic was identified by frontline staff, were found to be more successful than those topics ‘imposed’ on staff.

*Projects, where frontline staff identify the trigger, are the ones nurses are really interested in and commit to; compared to the knowledge focused triggers where we [nurse leaders] say what is needed. P4*

An enduring theme with all interviewees was the benefit that comes from a direct care nurse leading practice change. The rational proposed was that ‘bedside care providers are the ones that see the problems and are best placed to understand the ways to fix it’. This emancipatory bottom-up approach, where staff are encouraged to identify problems and empowered to fix them, was fundamental to practice change in the organisation.

*We shouldn’t underestimate the power of bottom-up [practice change] and the buy-in and the influence that happens naturally with it. People will do things for their colleagues that they wouldn’t be motivated to do if it came from the top down. P8*

Another highlighted strength of the model is its requirement for EBP initiatives to align to organisational priorities. This alignment was essential as ‘any practice change that is initiated by frontline staff must be supported by the organisation if it’s to be effective and sustainable’. It was recognised that when an EBP project did not align with a priority, there was a real risk that it would fail, potentially leaving the team disillusioned with the process.

*Aligning to organisational priorities is key. You can have the most brilliant idea in the world, but if you can’t get it through, then it’s meaningless. All that happens is people get frustrated and they won’t want to do it [change practice] again. P5*

An essential adjunct to the Iowa Model is the implementation guide [23] which helps users select implementation strategies that are appropriate for the stage of change and target group. When asked what the most useful part of the Iowa Model was, more than one participant stated that ‘the implementation guide is the most vital piece of the model’. The tool was developed after it was recognised that there was ‘very little guidance for staff on how to operationalise the implementation strategies’. The focus on implementation is acknowledged internally and externally as one of the useful features of the Iowa Model and a determinant of the local EBP environment.

### Support

Within the nursing directorate, EBP is ‘core business’ which is reflected in its visibility and status within the organisation. The Director of Nursing Research and EBP sits on the nursing and system executive team where she can influence the strategic direction and secure funds and resources necessary for EBP. There was an appreciation by participants that EBP is well supported with human, material, and financial resources. Mention was made of the Office of Nursing Research and EBP, which provides EBP education and training, supports staff undertaking EBP projects, and promotes EBP within and outside of the organisation.

*Having a centralised office is foundational to our EBP work. Our role is to have the EBP vision for the organisation and then provide the building blocks in order for us to get there. P9*

Continuous targeted education on EBP is a key feature of the organisation and one of the identified determinants of the EBP environment. EBP education is scaffolded, with staff gradually introduced to the concepts and methods in greater depth over time. New staff members are introduced to the Iowa Model at orientation; recent graduates are exposed further during their residency [24]; staff nurses attend grand rounds or the annual national EBP conference; new managers receive targeted education during induction; nurse specialist and leaders attend an intensive 3-day advanced EBP workshop [25]. This face-to-face education is supplemented with locally developed printed resource material, online modules, and an EBP textbook [6].

Theoretical education is complemented by experiential EBP training. This hands-on training includes an EBP Internship [26] and an EBP Change Champion program. Key features of both programs include training in EBP, dedicated project time, expert mentoring, and nurse manager support. The significant difference between the two programs is that the staff nurse chooses the internship topic while organisational leaders choose the change champion topic. The staff nurses interviewed particularly valued the sequestered time, while the managers appreciated that the projects were centrally funded, so they did not impact the unit staffing budget.

Nursing in the organisation has a strong academic partnership with the College of Nursing and other University of Iowa colleges. Participants saw a strong academic partnership as particularly important during the formation of the EBP environment. One interviewee who founded the Iowa Model recalled with appreciation how faculty helped ‘span the boundary between academia and practice’.

*They [nurse faculty] were generous with their time, they were comfortable working outside their specialty fields, and they appreciated the value of practice knowledge as much as theoretical knowledge. P5*

The academic and practice environments are highly intertwined with cross organisational representation on many committees. The synergy is further strengthened by the flow of students between the two organisations. Many staff study at the college, while many of the graduate students undertake EBP projects at the hospital. To ensure EBP projects target organisational priorities, the Nursing Research and EBP Committee maintains a list of priority topics and contacts.

The organisation is a university hospital, so it is well equipped with the knowledge infrastructure necessary for EBP. The hospital has a co-located medical library, and staff are well supported by a dedicated health librarian who is visible and accessible within the organisation. The Electronic Health Record (EHR) and the support within the organisation to leverage the EHR and digital data was reported as a facilitator of EBP. One participant described the benefit of using existing data in EBP projects.

*I always think about what data I get from what already exists, whether it be an ICD-9 code, procurement data, or [EHR] documentation. I don’t want a nurse to have to collect that information because that’s a waste of their time. P12*

The EHR was also seen as a valuable tool for implementing evidence into practice using evidence-based order sets, reminders, practice alerts, nursing documentation, and flow sheets.

### Facilitation

A significant proportion of the education, training, and support for EBP within the organisation is directed towards direct care nursing staff. Having direct care nurse leadership was reported to have several benefits: Frontline staff are acutely aware of clinical issues and opportunities for improvement; they have a patient-centred perspective; they understand workflows; they have established clinical networks and influence.

It was identified that staff nurses need to be surrounded by a supportive team, including an actively engaged nurse manager, to be successful at implementing evidence. There is an expectation in the organisation that the nurse manager of an area (i.e. units or clinics) will be an active member of a team to facilitate practice change [27]. As one participant put it, the nurse manager ‘makes or breaks an EBP project. If the manager doesn’t support it, then the project is going nowhere’. Nurse managers are also charged with maintaining a local climate that supports EBP. Interviewees said that good managers achieved this in several ways including sharing an EBP vision for the area, committing to developing their own and their staffs EBP competency, communicating the expectation of EBP, recognising staff for their EBP, and hiring staff that value EBP.

Nurse specialists facilitate EBP by acting as mentors for clinical staff undertaking projects and supporting managers to promote EBP in their area. As senior staff, they are well acquainted with the workings of the organisation, which makes them well placed to assist the project leads to connect with other departments and navigate the intricate governance process. One nurse specialist put it this way:

*So think of us as being there to help them [staff nurses] through the steps of the Iowa model. When they get stuck, they come to us, and we help them try to figure it out. P11*

Expert support from a nurse scientist is readily available. There is a specific role within the organisation that is dedicated to building EBP capacity and supporting staff undertaking EBP projects. Having an EBP expert on-hand provided staff with a sense of security, which was a significant benefit to both novice and experienced nurses alike.

*Well, I know that I can always reach out to [Nurse Scientist] and say, ‘Hey, I’m thinking about this. Here’s what my situation is, here’s what the problem is, this is what I’m finding’, and she would help walk me through it. P1*

### Context

The culture of nursing in the organisation is collaborative yet competitive. There is a high degree of staff cooperation, and it is common for staff to, ‘jump in to help support each other, knowing that this is what it takes to make projects successful’. The culture of collaboration is accompanied by a healthy dose of rivalry between and within the divisions. One participant viewed this rivalry as a positive driver of EBP.

*I think it’s our culture that you don’t want your division to look bad and have the other divisions in nursing doing things better. You want to make sure that your division is adequately represented and looks good compared to others. P2*

Nursing’s shared governance framework provides direct care nurses with the opportunity to be part of the decision-making process in the organisation [28]. Nursing EBP is overseen by the Nursing Research and EBP Committee, which is co-chaired by the Director of the Nursing Research and EBP, and a staff nurse. The shared governance structure facilitates communication and cooperation between the organisation’s committees and departments which is seen as essential for effective EBP.

*I think it [shared governance] is key. It’s about nurses being able to drive their own practice and our committee and our shared governance helps support this. You see, our EBP work impacts on the work of other committees, and other departments, so having this process that supports communicate is really beneficial. P8*

The organisation has a strong focus on *continuous improvement* where staff at all levels are permitted to question the status quo. In fact, direct care staff are challenged by senior staff to question work practices, and they are rewarded when they do [29]. One nurse recounted:

*I challenge my staff to ask themselves, ‘why are we doing it this way? Is there a better way to do it?’ We are trying to create this environment of, ‘could we be better?’ The status quo is not enough here. There’s always room for improvement. P12*

The interviewees acknowledged the relationship between EBP and quality improvement and the need for the two departments to work closely together. A nurse specialist whose role encompasses both described the essential interaction between the two within the organisation.

*So, as I see it ... All EBP is quality, but not all quality is EBP. In the Iowa Model we have a number of points where the two converge and diverge. At the front end of the model, we can have a trigger that might start an EBP project or a quality project depending on the circumstances. As we progress to implementation, we will often use quality methods to implement [and sustain] the change. Once we have implemented the change, we might hand off the project to quality for ongoing monitoring. P10*

The organisation highly values external recognition, and this is seen as a significant determinant of the EBP environment. The success of the EBP program has enhanced the organisation’s reputation, which is a source of pride to staff and leadership. The EBP projects are also a vital source of documentation for external benchmarking, accreditation, and award nominations. Many of the interviewees focused on the relationship between Magnet® and the organisation’s EBP program.

*I like to think that if we do it [EBP] for the right reasons, Magnet status will come. However, I will say that if we’re looking to ask why it’s a priority for the organisation, then we can link it to Magnet status. There’s a desire to have Magnet recognition, and so, that makes EBP a priority for the organisation. P7*

Leadership support was crucial for establishing and maintaining an EBP environment. A senior staff member described the period when the Iowa Model was first introduced as a ‘perfect storm’ of leadership. The hospital and the College of Nursing had leaders who shared a vision and were ‘open to the opportunity that evidence-based nursing practice promised’. It was acknowledged, however, that over the subsequent 25 years ‘some leaders hadn’t been as strong in terms of research and EBP’ but they had all valued the ‘improved clinical outcomes, the reduced costs, the awards and recognition’. These beneficial outcomes and the internal and external recognition were seen to reflect positively on the nursing leaders, which in turn bolsters support for the EBP program.

## Discussion

Having a standardised approach to EBP was recognised by participants as central to reliable, evidence-based care. This belief is also widely accepted in the literature [15], yet still, it is noted that EBP models are infrequently used, used superficially, or misused [5]. This shortcoming has led to a call to action for further wide-scale adoption and use of EBP models and more research to support them [10]. This research helps answer that call by providing valuable insights into how an organisation might take up, support, and sustain an EBP model.

Given that many of the EBP process models share similar steps, the most important consideration when selecting a model should be its acceptability to users. The Iowa Model was embraced by local staff for its focus on frontline practice issues and alignment to organisational priorities. In the literature, the perception of key stakeholders about whether a change is externally or internally driven is known to influence the success [30]. If the decision to adopt is made by leader edict with little user input, then implementation is less likely to succeed [31]. There is also evidence that a receptive context where the evidence-based change is congruent to the organisation’s mission and strategy is more likely to be effective [32].

Introducing an EBP model is an essential first step for achieving reliable, evidence-based care; however, to be most effective, the findings from this study suggest that it should be done in conjunction with optimisation of the practice environment. This finding is in keeping with the implementation science literature, which has long identified the influence of organisational and contextual factors on EBP. A recent systematic review of 36 studies found similar determinants to EBP as the ones identified in this research, including a supportive culture, effective networks and communication, leadership support, necessary resources, education and training, a focus on data and evaluation, and EBP champions [4]. This research adds to this body of knowledge by providing a detailed description of the determinants in one practice setting.

The relationship between outcomes and organisational and contextual factors is well described in the implementation science literature, but there is limited practical guidance on how best to optimise a practice environment for EBP. Currently, these factors are best described by determinant frameworks such as the Consolidated Framework for Implementation Research (CFIR) [33], or the Theoretical Domains Framework (TDF) [34], but these do not offer the step by step guidance required by clinicians. There are several hybrid process-determinant theories which address implementation along with organisational and contextual factors [35–37]. Organisations seeking to optimise the practice environment for EBP could use one of these hybrid models or pair an EBP process model with the CFIR or TDF.

Experiential programs where participants get the opportunity to acquire practical skills are a key feature of the EBP environment observed in this study. This approach to education aligns with current research on factors that impact nurses’ readiness for EBP. An integrative review of 39 studies on the topic found that regardless of the amount of EBP education received, the most positive predictor of evidence use was a nurse’s previous participation in EBP activities [38]. Organisations wishing to grow their EBP capacity should, therefore, consider practical hands-on training that enhances EBP competency [39].

Facilitation—defined as the act of enabling others to implement practice change—would seem to be a central yet almost invisible component of most EBP process models. A team-based facilitation approach was identified as one of the principal determinants of the local EBP environment. Team-based facilitation leverages different skill sets and professional networks to enable change at various organisational levels [40]. In this study, for example, the direct care nurses supported by the nurse manager use their insights into practice and influence over staff to shape practice at the micro-level (unit level), while the nurse specialists and nurse scientists used their professional networks and knowledge of the organisation to build the mezzo and macro-level support necessary to sustain and scale up the practice change.

### Strengths and limitations

Qualitative research is a valuable method for studying EBP because it rejects the notion of a single reality and instead appreciates the existence of multiple possibilities, which are context-bound, experientially based, and constructed through social interaction [14]. This in-depth interpretive study provides a detailed description of the determinants of the EBP environment in one practice setting, which may benefit similar organisations wishing to adopt an EBP model.

The focus of this research changed over time which may be seen as a limitation. However, this is in keeping with interpretive description, and indeed all qualitative methods [41]. If a question is worth studying qualitatively, it should be acknowledged that as it is investigated the emphasis of the inquiry may change as a deeper understanding of the phenomena is gained.

## Conclusion

The findings from this research support the idea that EBP is most effective in a supportive practice environment. In this setting, the determinants of the local EBP environment included a shared EBP model, education, hands-on training, knowledge infrastructure, team facilitation, and supportive culture and leadership. These findings will be of benefit to organisations and individuals wishing to implement an EBP model to improve the reliability and consistency of evidence-based care.

## Supplementary information

**Additional file 1:.** Semi-structured interview schedule.

## Data Availability

The data that support the findings of this study are available on request from the corresponding author [JD]. The data are not publicly available due to it containing information that could compromise participant privacy.

## Abbreviations

EBP: Evidence-based practice
CFIR: Consolidated Framework for Implementation Research
TDF: Theoretical Domains Framework
