# Supplementary material for: Determinants of an evidence-based practice environment: an interpretive description
Source: Implement Sci Commun. 2020 Oct 6;1:85. doi: 10.1186/s43058-020-00070-0 (PMC7542098; doi:10.1186/s43058-020-00070-0)
Supplement: Supplementary file 1 — Additional file 1:. Semi-structured interview schedule. [file 43058_2020_70_MOESM1_ESM.docx]

**Semi-Structured Interview Schedule**

1. What is your experience using the model?
2. What impact have you seen the model have on policy or practice?
3. How was this impact achieved?
4. When is the model most effective and when is it least effective?
5. Can you explain why the model might have different effects?
6. How does the practice environment influence the model's effectiveness?
7. What are the essential components of the model?
8. How were you introduced to the model?
9. How was the model introduced into your setting?
10. What factors influence the successful implementation of the model?
